# Mechanical Stress Induces Sodium Entry and Osmoprotective Responses in Murine Synovial Fibroblasts

**DOI:** 10.3390/cells13060496

**Published:** 2024-03-13

**Authors:** Annemarie Proff, Ute Nazet, Agnes Schröder, Jonathan Jantsch

**Affiliations:** 1 Institute for Medical Microbiology, Immunology, and Hygiene, Center for Molecular Medicine Cologne (CMMC), University Hospital Cologne and Faculty of Medicine, University of Cologne, 50935 Cologne, Germany; jonathan.jantsch@uk-koeln.de; 2 Department of Orthodontics, University Hospital Regensburg, 93053 Regensburg, Germanyagnes.schroeder@ukr.de (A.S.); 3 Institute for Medical Microbiology and Hygiene, University Hospital Regensburg, 93053 Regensburg, Germany

**Keywords:** synovial fibroblast, sodium chloride, osteoarthritis

## Abstract

Osteoarthritis (OA) is a multifactorial disease depending on molecular, genetic, and environmental factors like mechanical strain. Next to the cartilage and the subchondral bone, OA also affects the synovium, which is critically involved in the maintenance of joint homeostasis. As there is a correlation between the extracellular sodium content in the knee joint and OA, this study investigates the impact of sodium on OA-associated processes like inflammation and bone remodeling without and with mechanical loading in synovial fibroblasts. For that purpose, murine synovial fibroblasts from the knee joint were exposed to three different extracellular sodium chloride concentrations (−20 mM, ±0 mM and +50 mM NaCl) in the absence or presence of compressive or intermittent tensile strain. In addition to the intracellular Na^+^ content and gene expression of the osmoprotective transcription factor nuclear factor of activated T cells 5 (*Nfat5*), the gene and protein expression of inflammatory mediators (interleukin-6 (IL6), prostaglandin endoperoxide synthase-2 (*Ptgs2*)/prostaglandin E_2_ (PGE_2_)), and factors involved in bone metabolism (receptor activator of NF-κB ligand (RANKL), osteoprotegerin (OPG)) were analyzed by qPCR and ELISA. Mechanical strain already increased intracellular Na^+^ and *Nfat5* gene expression at standard salt conditions to levels obtained by exposure to increased extracellular Na^+^ content. Both high salt and compressive strain resulted in elevated IL6 and PGE_2_ release. Intermittent tensile strain did not increase *Il6* mRNA expression or IL6 protein secretion but triggered *Ptgs2* expression and PGE_2_ production. Increased extracellular Na^+^ levels and compressive strain increased RANKL expression. In contrast, intermittent tension suppressed RANKL expression without this response being subject to modification by extracellular sodium availability. OPG expression was only induced by compressive strain. Changes in extracellular Na^+^ levels modified the inflammatory response and altered the expression of mediators involved in bone metabolism in cells exposed to mechanical strain. These findings indicate that Na^+^ balance and *Nfat5* are important players in synovial fibroblast responses to mechanical stress. The integration of Na^+^ and Na^+^-dependent signaling will help to improve the understanding of the pathogenesis of osteoarthritis and could lead to the establishment of new therapeutic targets.

## 1. Introduction

Millions of people worldwide suffer from osteoarthritis (OA), a chronic degenerative joint disease [1,2]. It can be accompanied by severe pain and immobilization, reducing the quality of life of the patients [3]. There is still no established curative therapy available yet [1,4,5]. The knee joint is one of the joints most frequently affected by OA, as it is exposed to severe mechanical stress in everyday life. OA of the knee joint is a significant orthopedic problem and is associated with high costs in the healthcare system [6,7,8]. The disease is multifactorial: genetic, epigenetic, and environmental factors contribute to the development and progression of OA [9,10,11,12]. Various joint structures, including the cartilage and synovium, are significantly involved in the development and progression of this disease [13]. The synovial fibroblasts investigated in this study represent an important cell type of the synovium as they are involved in the maintenance of joint homeostasis [14,15]. In OA patients, there is inflammation of the synovium, which is promoted by increased cartilage attrition [11,16,17]. The inflammatory and bone remodeling processes involved in this process are subject to various influencing factors. The factor of mechanical stress has already been linked to the development of OA in previous studies. While moderate mechanical stress is essential for joint preservation, exceptionally high levels of non-physiological and inefficient mechanical stress are associated with disease-promoting processes [12,18,19].

It is well established that various molecular mechanisms contribute to the development and progression of OA [20]. The remodeling of extracellular matrix components, inflammation, and bone metabolism are important modulatory factors. Previous studies show a clear relationship between elevated interleukin 6 (IL6) levels in the blood and tissue and the incidence of the development of OA [21,22,23]. Furthermore, IL6 was reported to be involved in the degradation of cartilage [24,25,26]. Prostaglandin E_2_ (PGE_2_) is synthesized by prostaglandin endoperoxide synthase 2 (PTGS2) and is one of the main catabolic factors involved in OA by critically contributing to the degradation of cartilage [27]. Moreover, subchondral bone osteoblasts from OA patients display high levels of the osteoclast-promoting factor receptor activator of NF-κB ligand (RANKL) and low levels of its decoy receptor osteoprotegerin (OPG) [28,29]. In the synovial fluid of late-stage OA patients, the RANKL/OPG-ratio is increased, indicating propagated osteoclastogenesis, which ultimately promotes subchondral bone resorption and bone loss [30].

The expression of IL6, PTGS2/PGE_2_ as well as OPG was shown to be regulated by the osmoprotective transcription factor nuclear factor of activated T cells 5 (NFAT5) [31,32,33,34]. Since increased extracellular ion abundance and tonicity regulate NFAT5 expression, the surrounding ionic microenvironment may affect the cellular responses induced by mechanical stress. Changes in tissue sodium concentration in response to a high-salt diet [30,31] or inflammation [32,33] influence numerous processes at the molecular level, indicating a key role of salt balance for different cell types [34]. Extracellular Na^+^ content in tissues can trigger the expression of the osmoprotective transcription factor NFAT5 [31,32,33,34]. Moreover, NFAT5 expression is increased after mechanical loading in fibroblasts [35]. Sodium MRI studies have shown a correlation between the extracellular sodium concentrations and OA-characteristic catabolic remodeling processes of the extracellular matrix [36]. The osmoprotective transcription factor NFAT5 plays a decisive regulatory role in connection with extracellular sodium levels. Changes in tonicity increase NFAT5 expression and trigger various different regulatory mechanisms [37,38,39,40].

The aim of this study was to analyze the relationship between different mechanical loading protocols, extracellular sodium chloride concentrations, and inflammatory and bone remodeling processes, which also play a critical role during OA pathogenesis in synovial fibroblasts.

## 2. Materials and Methods

### 2.1. Cell Culture Experiments

#### 2.1.1. General Cell Culture Conditions

Primary, murine synovial fibroblasts from the knee joints (passages four to eight; Supplemental Figure S1a) of eight-week-old wildtype BL/6 mice male mice were used. The mice were euthanised and dissected conforming to national and institutional regulations. The cells have been isolated and were characterized previously [41]. The synovial fibroblasts were cultured at 37 °C in RPMI1640 with GlutaMAX™ (61870-010, Gibco, Thermo Fisher Scientific, Waltham, MA, USA) containing 10% fetal calf serum (FCS, P30-3302, PAN-Biotech, Aidenbach, Germany), 1% antifungal/antibiotic (A5955, Sigma-Aldrich, Darmstadt, Germany), and 1% ascorbic acid (A8960, Sigma-Aldrich).

#### 2.1.2. Experiments with Different Salt Concentrations without Mechanical Loading

Inflammation and diet can affect sodium content in different ways [36,37,38,42]. To avoid any cytotoxic effects, different lower and higher NaCl concentrations were tested, and concentrations without increased LDH release were used (Supplemental Figure S1b). The experiments were performed with three different extracellular sodium chloride concentrations in the following media: a low salt medium (−20 mM Na^+^ compared to standard salt medium; resulting in a total Na^+^ content of 125 mM), a standard salt medium (±0 mM Na^+^ compared to standard salt medium; resulting in a total Na^+^ content of 145 mM), and a high salt medium (+50 mM Na^+^ compared to standard salt medium; resulting in a total Na^+^ content of 195 mM). To prepare the media with the different salt concentrations, the culture medium was mixed with either sterile, deionized water (L0015, Biochrom, Cambridge, UK), the same amount of saline solution (0.9% NaCl), or sodium chloride supplemented with a 2.5 M NaCl stock solution (3957.1, Carl Roth, Karlsruhe, Germany). The final Na^+^ concentrations in the medium were confirmed by atomic adsorption spectroscopy. For the experiments, a total of 35,000 synovial fibroblasts were seeded per well of a 24-well plate and incubated overnight at 37 °C. After that, the medium was changed, and the synovial fibroblasts were incubated for another 48 h. 

#### 2.1.3. Experiments with Static Compressive Force Application

**Figure 1:**
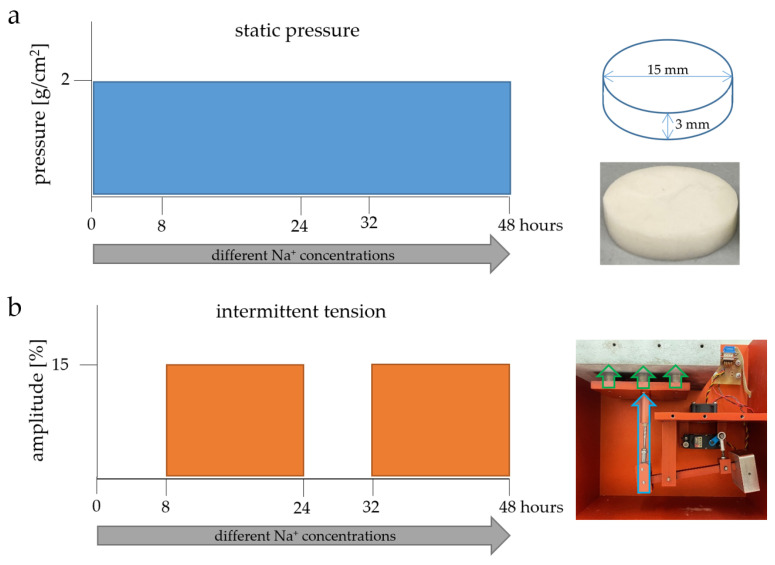
Schematic representation of the performed experiments. Mouse synovial fibroblasts from knee joints were incubated in different Na^+^ concentrations (−20 mM, ±0 mM, +50 mM) for a total of 48 h. For compressive force treatment, a sterile ZnO_2_ plate (2 g/cm^2^), as illustrated on the right side, was placed on the synovial fibroblasts for 48 h (**a**). Intermittent cyclic tension was performed for at least 48 h with two cycles of 8 h at a 0% amplitude followed by 16 h at a 15% amplitude using a cell stretching machine (**b**). The piston (marked with the blue arrow) pushes the stamps (green arrows) upwards according to the specified frequency and amplitude. The light gray metal block contains a collagen-coated 6-well Bioflex plate with a flexible underside.

A total of 35,000 synovial fibroblasts were seeded per well of a 24-well plate and incubated overnight at 37 °C. Then, cells were subjected to three different sodium chloride concentrations (−20 mM, ±0 mM, +50 mM) for 48 h. During the incubation period, the cells were exposed to compressive force using ZnO_2_ plates (2 g/cm^2^; Figure 1a) [43].

#### 2.1.4. Experiments with Intermittent Tensile Strain

A total of 200,000 primary synovial knee fibroblasts were seeded per well on collagen-coated 6-well Bioflex plates (BF-3001C, Dunn Labortechnik, Asbach, Germany) and incubated overnight at 37 °C. On the following day, the culture medium was replaced by a medium with different sodium chloride concentrations (−20 mM, ±0 mM, +50 mM). Then, synovial fibroblasts were subjected to intermittent tensile strain (amplitude 15%; frequency 0.5 Hz; two eight-hour rest periods followed by 16 h of stretching; Figure 1b) [41,44,45]. The synovial fibroblasts were exposed to different sodium chloride concentrations for a total of 48 h.

### 2.2. Assessment of Intracellular Na^+^ Using Atomic Adsorption Spectrometry

Essentially, detection of intracellular Na^+^ was performed as described earlier [46,47]. The supernatant was removed, and the cells were washed three times with a sucrose solution (34.24%; 4621.2 Carl Roth, Karlsruhe, Germany). Synovial fibroblasts were lysed by incubation in 0.1% Triton X-100 (327371000, Acros Organics, Antwerp, Belgium) for 20 min. The cell lysates were scraped off with a cell scraper and transferred to reaction tubes. To quantify sodium in the lysate, atomic absorption spectrometry (iCE 3500, Thermo Fisher Scientific, Waltham, MA, USA) was used. For this purpose, 100 μL of the lysates were added to a 3 mL dilution solution (0.1% HNO_3_ (X898.2, Carl Roth), 0.5% CsCl solution (289329, Sigma-Aldrich, Darmstadt, Germany) in H_2_O_dd_) and mixed by inverting. Standard dilution series of Na^+^ (2337, Carl Roth) were prepared within the linear range.

### 2.3. Quantitative Polymerase Chain Reaction (qPCR)

The qPCR protocol basically followed the previously published proceedings [41,44]. Briefly, RNA was extracted using RNA-Solv (R6830-01, VWR, Darmstadt, Germany) according to the manufacturer’s protocol. A concentration specific amount of RNA was mixed with nuclease-free water (T143.5, Carl Roth) for cDNA synthesis. A total of 4.5 μL of master mix, consisting of 2 µL of M-MLV buffer (M531A, Promega, Madison, WI, USA) and 0.5 µL each of Oligo_dT_ (SO132, Thermo Fisher Scientific), Random Hexamer Primer (SO142, Thermo Fisher Scientific), dNTPs (L785, Carl Roth), RNase Inhibitor (EO0381, Thermo Fisher Scientific) and Reverse Transcriptase (M170B, Promega), were added per sample. The samples were incubated for one hour at 37 °C in a thermocycler (Biometra, Analytik Jena, Jena, Germany), followed by heating to 95 °C for 2 min. A total of 1.5 μL of each diluted cDNA sample was pipetted into a nuclease-free 96-well plate (712282, BiozymScientific, Hessisch Oldendorf, Germany) in duplicate. To each well, 8.5 μL of a master mix consisting of 0.25 μL forward primer (Table 1), 0.25 μL reverse primer (Table 1), 5 μL Luna Universal qPCR Mix (M3003E, New England Biolabs, Frankfurt am Main, Germany), and 3 μL nuclease-free water (T143.5, Carl Roth) was added. The 96-well plate was then sealed (712350, Biozym) and centrifuged. The qPCR was performed using a Realplex2 cycler (Eppendorf, Hamburg, Germany). *Hprt* and *Sdha* were used as reference genes (Table 1). All primers were designed for exon-intron spanning. Relative gene expression was determined using the 2^−ΔCT^ formula with ΔCT = C_q_(target gene) − C_q_ (geometric mean *Hprt*/*Sdha*) [48,49]. It was then divided by the arithmetic mean of the control group to obtain relative gene expression with respect to the control group.

### 2.4. Enzyme Linked Immune Absorbent Assays (ELISAs)

All ELISAs were performed according to the manufacturer’s instructions. The following ELISA kits were used: interleukin 6 (MyBioSource, San Diego, CA, USA, Murine IL6 ELISA, MBS335514), prostaglandin E_2_ (MyBioSource, Mouse Prostaglandin E_2_ ELISA, MBS266212), osteoprotegerin (Thermo scientific, Mouse OPG (TNFRSF11B) ELISA Kit, EMTNFRSF11B) and RANKL (Thermo scientific, Mouse TRANCE (TNFSF11) ELISA Kit, EMTNFSF11).

### 2.5. Statistics

Statistical analysis was performed using the program GraphPadPrism 9.5. Before statistical evaluation, all absolute data values, except the protein data of the ELISA, were divided by the respective arithmetic mean of the control group without mechanical stress to obtain normalized data values. The bars show the mean, and the horizontal lines the standard error of the mean. The normal distribution of the data was examined with the Shapiro–Wilk test. The homogeneity of the groups was determined using the Brown-Forsythe test. Depending on the normal distribution and homogeneity of the data, a Welch-corrected ANOVA with Dunnett’s T3 multiple comparisons test was performed, and differences were considered statistically significant at *p* < 0.05.

## 3. Results

### 3.1. Impact of Different Extracellular Na^+^ Concentrations on Synovial Fibroblasts

**Figure 2:**
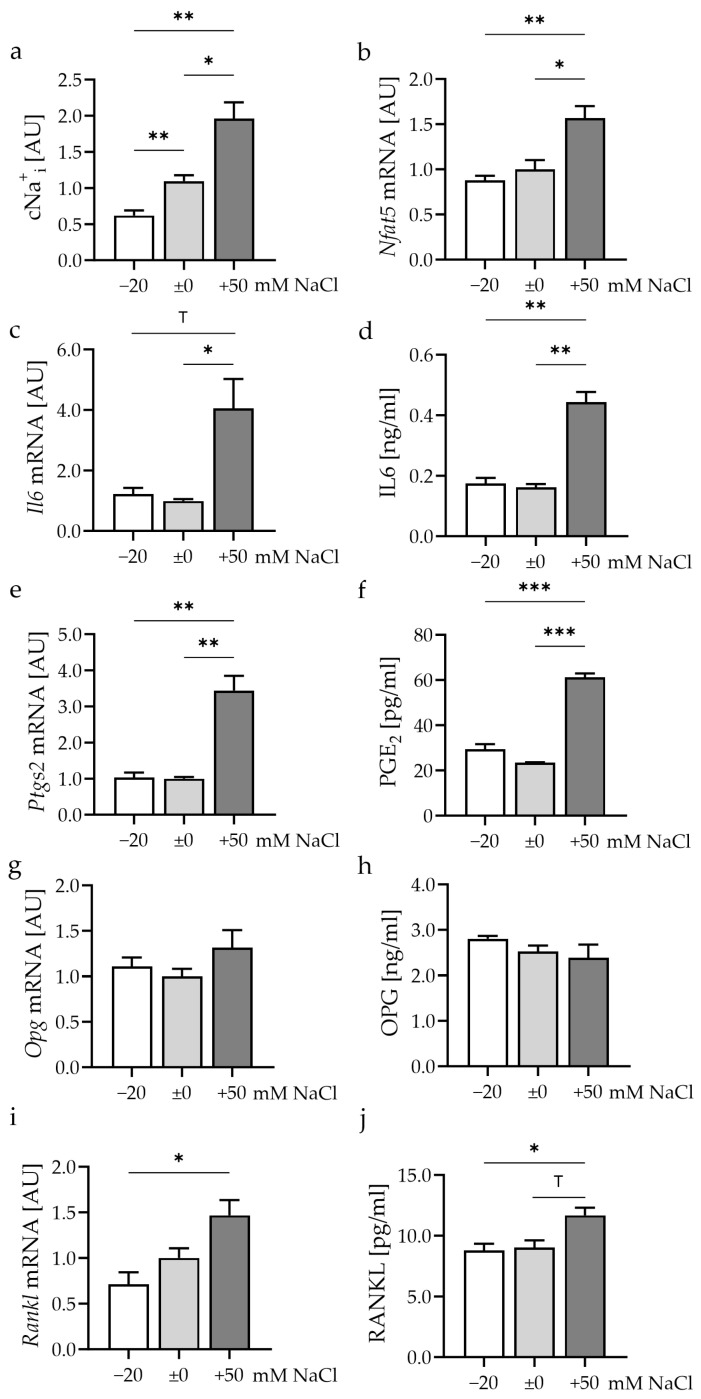
Impact of different extracellular NaCl concentrations for 48 h on relative Na^+^_i_ content ((**a**); *n* = 7), *Nfat5* mRNA (**b**), *Il6* mRNA (**c**) and IL6 protein secretion (**d**), *Ptgs2* mRNA (**e**) and PGE_2_ secretion (**f**), *Opg* mRNA (**g**) and OPG protein secretion (**h**), as well as *Rankl* mRNA (**i**) and RANKL protein secretion (**j**). mRNA expression: *n* ≥ 7; protein secretion: *n* = 4. Statistics: Welch-corrected ANOVA with Dunnett’s T3 multiple comparisons test; ^T^ *p* < 0.1; * *p* < 0.05; ** *p* < 0.01; *** *p* < 0.001.

First, the effects of different extracellular salt concentrations on intracellular Na^+^ (Na_i_^+^) without any additional mechanical loading were analyzed. The reduction of extracellular Na^+^ by −20 mM or addition of +50 mM NaCl did not induce any cytotoxicity (Supplemental Figure S1b). The reduction of extracellular Na^+^ (Na_e_^+^) by −20 mM decreased intracellular Na^+^ (Na_i_^+^, *p* = 0.03; Figure 2a), while increasing Na_e_^+^ by 50 mM increased Na_i_^+^ levels (*p* = 0.021). Gene expression of the osmoprotective transcription factor nuclear factor of activated T cells 5 (*Nfat5*) was increased after incubation in salt-rich conditions (*p* ≤ 0.014; Figure 2b). NFAT5 can regulate the expression of the inflammatory genes interleukin-6 (*Il6*) and the inflammatory enzyme gene prostaglandin endoperoxide synthase-2 (*Ptgs2*) [31,32,34]. Accordingly, high salt conditions increased *Il6* gene expression (*p* = 0.046; Figure 2c) and IL6 protein release (*p* = 0.004; Figure 2d). The mRNA expression of *Ptgs2* (*p* = 0.002; Figure 2e) and the secretion of PGE_2_ (*p* < 0.001; Figure 2f) were also upregulated with increased extracellular NaCl content. Expression of the bone protective decoy receptor osteoprotegerin (OPG) was not affected on either the mRNA (*p* ≤ 0.406; Figure 2g) or the protein level (*p* ≤ 0.509; Figure 2h) by exposure to high salt conditions. Receptor activator of NF-κB ligand (RANKL) mRNA expression (*p* = 0.013; Figure 2i) and protein secretion (*p* = 0.013; Figure 2j) were increased by the addition of salt compared to the low salt group. Increased *Il6*/IL6, *Ptgs2*/PGE_2_, and *Rankl*/RANKL expression/release under high salt conditions may point towards the induction of osteoclastogenesis by murine knee synovial fibroblasts upon exposure to high salt conditions.

### 3.2. Impact of Static Pressure Application and Extracellular Na^+^ Levels on Synovial Fibroblasts

**Figure 3:**
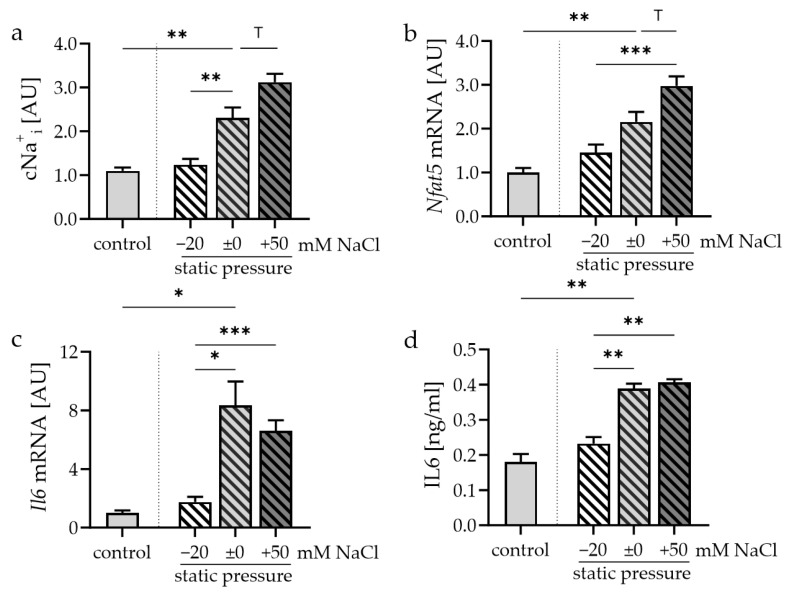

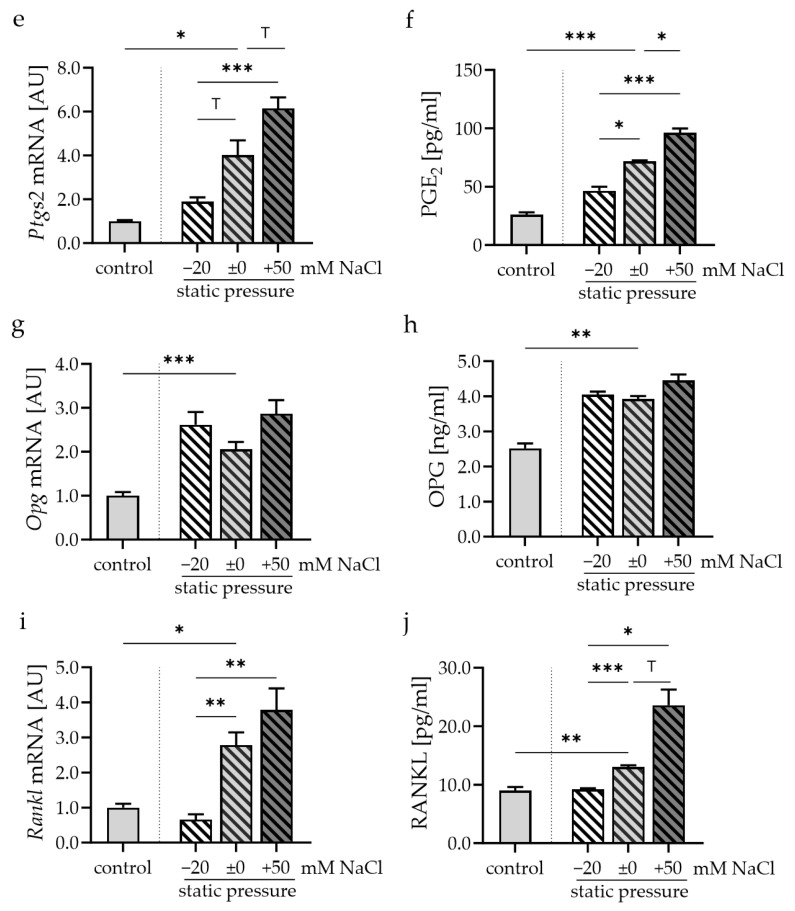
Impact of different extracellular NaCl concentrations during static pressure application on relative Na^+^_i_ ((**a**); *n* = 7), *Nfat5* (**b**), IL6 mRNA (**c**) and protein secretion (**d**), *Ptgs2* mRNA (**e**) and PGE_2_ secretion (**f**), OPG mRNA (**g**) and protein secretion (**h**), as well as RANKL mRNA (**i**) and protein secretion (**j**). mRNA expression: *n* ≥ 7; protein secretion: *n* = 4. Statistics: Welch-corrected ANOVA with Dunnett’s T3 multiple comparisons test; ^T^ *p* < 0.1; * *p* < 0.05; ** *p* < 0.01; *** *p* < 0.001.

Na_e_^+^ levels had no effects on cytotoxicity during static pressure (Supplemental Figure S1c). After static pressure application in medium with different extracellular salt contents, Na_i_^+^ was measured. Na_i_^+^ increased after compressive strain without the external addition of NaCl (*p* = 0.005; Figure 3a). Of note, the lowering of Na_e_^+^ was accompanied by lower Na_i_^+^ concentrations (*p* = 0.008), while increasing Na_e_^+^ levels only tended to increase Na_i_^+^ levels (*p* = 0.061; Figure 3a). *Nfat5* gene expression was increased by static pressure (*p* = 0.004) and correlated with external NaCl levels in cells exposed to static pressure (*p* < 0.001; Figure 3b).

*Il6* mRNA expression (*p* = 0.011; Figure 3c) and IL6 protein secretion (*p* = 0.002; Figure 3d) were elevated in reaction to static pressure application. The reduction of extracellular NaCl by −20 mM decreased pressure-induced *Il6* gene (*p* = 0.016) and protein expression (*p* = 0.003). Under high salt conditions, there was no further detectable increase in *Il6* expression or release (Figure 3c,d). Compressive strain increased *Ptgs2* mRNA (*p* = 0.010; Figure 3e). Additional extracellular NaCl tended to further elevate *Ptgs2* gene expression after static pressure application (*p* = 0.087), while an additional reduction in extracellular sodium chloride content reduced *Ptgs2* mRNA (*p* = 0.055; Figure 3e). Compressive force application increased PGE_2_ secretion (*p* < 0.001; Figure 3f). This response was further increased by additional exposure to high extracellular NaCl conditions (*p* ≤ 0.023). 

*Opg* mRNA was elevated after compression of synovial fibroblasts (*p* < 0.001; Figure 3g). No significant additional effect of extracellular NaCl on *Opg* gene expression was detectable in cells exposed to static force. These effects were also mirrored at the protein level (Figure 3h). Compressive strain increased *Rankl* gene expression (*p* = 0.012; Figure 3i) and RANKL protein secretion (*p* = 0.007; Figure 3j). In cells treated with compressive static strain, additional extracellular NaCl further increased RANKL protein secretion (Figure 3i,j).

### 3.3. Impact of Intermittant Tension and Extracellular Na^+^ on Synovial Fibroblasts

**Figure 4:**
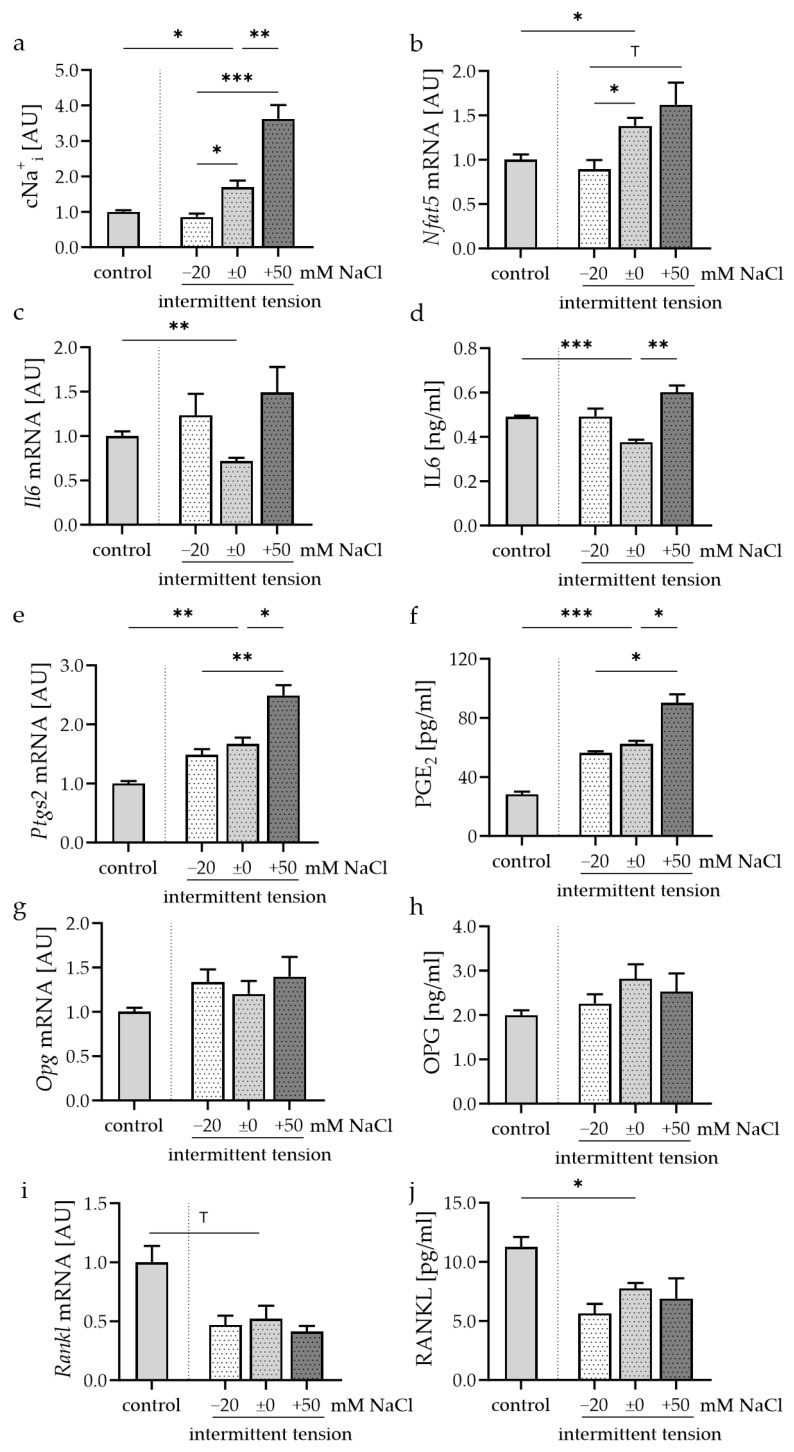
Impact of different extracellular Na^+^ concentrations during intermittent tension on Na_i_^+^ level ((**a**); *n* = 8), *Nfat5* (**b**), IL6 mRNA (**c**) and protein secretion (**d**), *Ptgs2* mRNA (**e**) and PGE_2_ secretion ((**f**) OPG mRNA (**g**) and protein secretion (**h**), as well as RANKL mRNA (**i**) and protein secretion (**j**). mRNA expression: *n* ≥ 7; protein secretion: *n* = 4. Statistics: Welch-corrected ANOVA with Dunnett’s T3 multiple comparisons test; ^T^ *p* < 0.1; * *p* < 0.05; ** *p* < 0.01; *** *p* < 0.001.

Like static compressive strain, intermittent tension did not exert any cytotoxicity (Supplemental Figure S1d). Intermittent compressive strain increased Na_i_^+^ content (*p* = 0.028; Figure 4a) and *Nfat5* expression (*p* = 0.023; Figure 4b). Under these conditions, Na_i_^+^ levels depended on extracellular NaCl availability (*p* ≤ 0.011; Figure 4a). A reduction in extracellular salt led to diminished *Nfat5* mRNA after tensile strain (*p* = 0.014), while there was no significant effect on *Nfat5* levels in cells treated with intermittent compressive strain after increasing extracellular NaCl levels (*p* = 0.838; Figure 4b).

In contrast to static compressive strain, intermittent tension reduced *Il6* mRNA (*p* = 0.004; Figure 4c) and IL6 protein secretion (*p* < 0.001; Figure 4d). While extracellular NaCl had no statistically significant impact on *Il6* mRNA gene expression after intermittent tensile loading, increased extracellular NaCl concentrations elevated IL6 protein secretion significantly (*p* = 0.007; Figure 4d). Intermittent tension induced *Ptgs2* gene expression and PGE_2_ secretion (*p* ≤ 0.002; Figure 4e,f). Increases in extracellular NaCl boosted this effect (*p* ≤ 0.033). 

Gene and protein expression of the RANKL decoy receptor OPG were neither affected by intermittent tension nor by increased or reduced extracellular NaCl content (Figure 4g,h). Intermittent tension reduced *Rankl* mRNA levels (*p* = 0.067; Figure 4i) and RANKL secretion (*p* = 0.049; Figure 4j). This response to intermittent tension was not modified by extracellular NaCl availability (Figure 4i,j).

## 4. Discussion

Changes in intra- and extracellular ion concentrations influence numerous processes at the molecular level [50,51,52]. A low-salt diet is generally recommended for a healthier lifestyle [53,54]. The progression and pathogenesis of knee OA might be influenced by tissue Na^+^ content, as a high-salt diet in particular is associated with OA-promoting inflammatory processes [55,56].

The inflammatory mediators IL6, PTGS2, and PGE_2_ have already been described in connection with OA in relation to catabolic processes [24,25,26,27,57]. An increased secretion of PGE_2_ was demonstrated in murine synovial fibroblasts of the temporomandibular joint and osteoblasts of the subchondral bone exposed to mechanical stress [44,57,58]. Increased expression of inflammatory factors occurs particularly during short periods of intense mechanical stress, similar to the initial stage of OA. During long-term stress, inflammatory processes are less dominant than remodeling processes of the extracellular matrix [58]. Contrary to *Ptgs2*/PGE_2_, there were different expression patterns for IL6 that were detectable depending on the nature of the applied force. With compressive strain, there was an increase in *Il6* gene expression and IL6 protein secretion, while expression decreased with intermittent tension. These findings are in line with the literature [43,44,58].

In contrast to the inflammatory processes investigated, the bone remodeling factor OPG was unaffected by a high salt exposure. Rather, the mechanical load plays a decisive role. Increased RANKL and decreased OPG levels are both associated with catabolic bone remodeling processes [28,30,59]. Increased RANKL was detected in the synovial fluid of patients with temporomandibular joint OA [60] and in the serum of patients with knee OA [30]. Confirming earlier studies [44], it was found that only compressive force application increased expression of RANKL but not intermittent tensile strain. 

Exposure to high extracellular Na^+^ levels triggers increased intracellular Na^+^ concentrations [61]. In line with this, exposure to high extracellular NaCl resulted in higher intracellular Na^+^ levels in mouse fibroblasts as well. Here it was shown that mechanical stress can induce increases in intracellular Na^+^ under normal cell culture conditions in mouse fibroblasts. This suggests that increases in Na^+^ might be involved in signal transduction and cellular responses to mechanical stress. In addition to mechanostress, hypoxia is able to trigger increases in Na^+^ levels in mitochondria, which ultimately interfere with electron transfer and mitochondrial energy generation. Therefore, it was suggested that intracellular Na^+^ might act as a second messenger [62].

Of note, in this study, intracellular Na^+^ levels were correlated with the expression of the transcription factor *Nfat5*, which is not only involved in various downstream osmoprotective but also inflammatory [34,39] and bone remodeling processes [33,34,63]. NFAT5 promotes the expression of inflammatory mediators in various tissues [34,39]. Increased salt concentrations and subsequently elevated NFAT5 expression were associated with increased activation of proinflammatory macrophages and T cell outputs [37,38,42,64]. NFAT5 could also play a significant role as an intermediate mediator in OA-associated inflammatory processes. For instance, Yoon et al. reported an upregulation of NFAT5 under proinflammatory conditions in rheumatoid arthritis [65]. The interdependence of NFAT5 and inflammation is also important. In line with this notion, NFAT5 can be upregulated not only by osmotic stress but also by inflammatory cytokines [66]. Although both mechanical loading protocols increase intracellular Na^+^ concentrations and NFAT5 expression, the effects on inflammatory and bone remodeling genes are not uniform.

In this context, it should be kept in mind that, next to its effect on NFAT5 expression, Na^+^ itself might represent a second messenger affecting many cellular signaling processes and inflammatory responses [50,62]. Of note, increasing intracellular Na^+^ levels is not sufficient to mimic effects induced by exposure to increases in high extracellular salt in macrophages, but additional signals, such as hypertonic membrane stress, are required [46]. 

In this study, for instance, intracellular Na^+^ was correlated with RANKL expression after compressive strain, but there was no correlation upon exposure to intermittent tensile strain. Next, although OPG was reported to be a NFAT5 target gene in osteoblasts [33], no effects of either mechanical loading or salt concentration on *Opg* mRNA expression or OPG protein level were observed in murine knee synovial fibroblasts. Therefore, it is very tempting to speculate that there is a complex and context-dependent interplay of intracellular Na^+^ levels, transcription factor abundance, and membrane responses that ultimately drive the outputs of cells. If and how our findings on inflammatory and bone remodeling outputs are mechanistically linked to intracellular Na^+^ concentrations and *Nfat5* need further experimental clarification. This could be particularly interesting because local ion concentrations in the joint could be influenced therapeutically, for example, through joint injections [67].

This study has several limitations that should be mentioned. OA is a multifactorial disease that depends on various cell types. This study only investigated synovial fibroblasts. The suggested relationship with the pathophysiology of osteoarthritis is correlative and must be validated in further mechanistic experiments.

## 5. Conclusions

Overall, the data of this in vitro study indicate that extracellular sodium chloride concentrations impact synovial fibroblast responses upon mechanical strain. Of utmost interest, however, our data show that mechanical stress results in enhanced Na_i_^+^ and *Nfat5* levels even under normal salt conditions. This suggests that Na_i_^+^ and *Nfat5* could play an important role in transducing mechanostress signals to cells.

## Figures and Tables

**Table 1:** Reference (*Hprt* and *Sdha*) and target gene primers used for qPCR.

Gene	Gene Name	5′-Forward-Primer-3′	5′-Reverse-Primer-3′
*Hprt*	hypoxanthine guanine phosphoribosyl transferase	AGCTTGCTGGTGAAAAGGAC	AGTCAAGGGCATATCCAACAAC
*Il6*	Interleukin-6	AAAGCCAGAGTCCTTCAGAGAG	CCTTAGCCACTCCTTCTGTGAC
*Nfat5*	nuclear factor of activated T-cells	AAATGACCTGTAGTTCTCTGCTTC	GCTGTCGGTGACTGAGGTAG
*Opg*	osteoprotegerin	CCTTGCCCTGACCACTCTTAT	CACACACTCGGTTGTGGGT
*Ptgs2*	prostaglandin endoperoxide synthase-2	TCCCTGAAGCCGTACACATC	TCCCCAAAGATAGCATCTGGAC
*Rankl*	receptor activatior of NF-κB ligand	AAACGCAGATTTGCAGGACTC	CCCCACAATGTGTTGCAGTTC
*Sdha*	succinat dehydrogenase complex, subunit A	AACACTGGAGGAAGCACACC	AGTAGGAGCGGATAGCAGGAG

## Data Availability

All data are available on request.

## Supplementary Materials

The following supporting information can be downloaded at: https://www.mdpi.com/article/10.3390/cells13060496/s1, Figure S1: Primary murine synovial fibroblasts form the knee joint (a). Lactat dehy-drogenase (LDH) release of murine synovial fibroblasts exposed to different NaCl concentriations without mechanical loading (b; *n* = 4), static compressive force (c; *n* = 9) and intermittent tension (d; *n* = 6). The LDH assay was performed according to the manufacturer’s instructions (04744926001, Roche, Mannheim, Germany). Statistics: Welch-corrected ANOVA with Dunnett´s T3 multiple comparisons test; * *p* < 0.05; ** *p* < 0.01.
