# Supplementary material for: Mechanical Stress Induces Sodium Entry and Osmoprotective Responses in Murine Synovial Fibroblasts
Source: Cells. 2024 Mar 13;13(6):496. doi: 10.3390/cells13060496 (PMC10969659; doi:10.3390/cells13060496)
Supplement: Supplementary file 1 [file cells-13-00496-s001.zip › cells-2809353-supplementary.pdf]

cells

Article

Mechanical Stress Induces Sodium Entry and Osmoprotective Responses in Murine Synovial Fibroblasts

Annemarie Proff [1,*], Ute Nazet [2], Agnes Schröder [2,3] and Jonathan Jantsch [1]

[1] Institute for Medical Microbiology, Immunology, and Hygiene, Center for Molecular Medicine Cologne (CMMC), University Hospital Cologne and Faculty of Medicine, University of Cologne, 50935 Cologne, Germany; aproff@smail.uni-koeln.de (AP); jonathan.jantsch@uk-koeln.de (JJ)
[2] Department of Orthodontics, University Hospital Regensburg, 93053 Regensburg, Germany; ute@nazet.de (UN); agnes.schroeder@ukr.de (AS);
[3] Institute for Medical Microbiology and Hygiene, University Hospital Regensburg; 93053 Regensburg, Germany

* Correspondence: aproff@smail.uni-koeln.de (AP)

5. Supplemental Figure

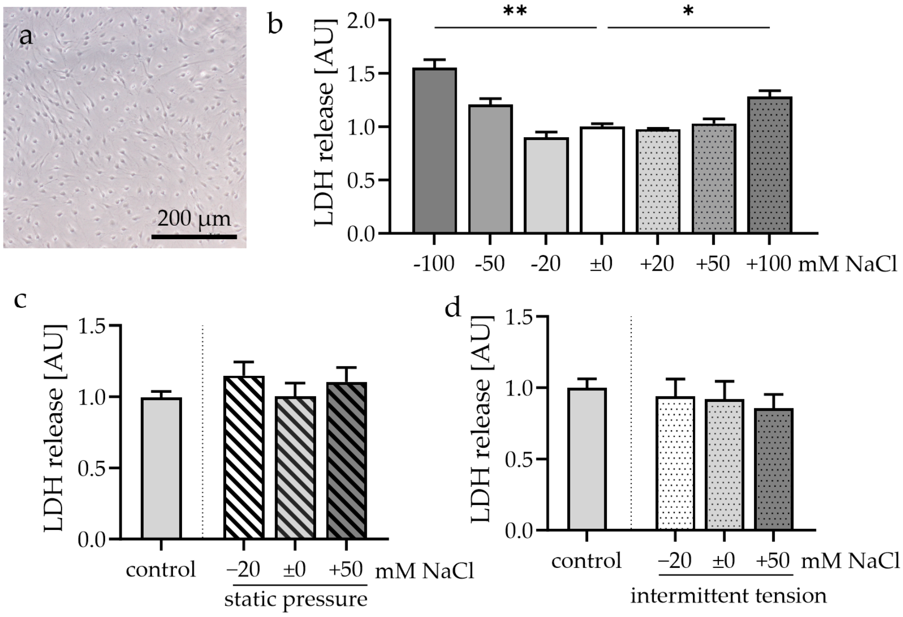

Supplemental Figure S1: Primary murine synovial fibroblasts form the knee joint (a). Lactat dehydrogenase (LDH) release of murine synovial fibroblasts exposed to different NaCl concentriations without mechanical loading (b; n=4), static compressive force (c; n=9) and intermittent tension (d; n=6). The LDH assay was performed according to the manufacturer's instructions (04744926001, Roche, Mannheim, Germany). Statistics: Welch-corrected ANOVA with Dunnett´s T3 multiple comparisons test; *P<0.05; **P<0.01.
